# Alterations of Cardiovascular Complexity during Acute Exposure to High Altitude: A Multiscale Entropy Approach

**DOI:** 10.3390/e21121224

**Published:** 2019-12-15

**Authors:** Andrea Faini, Sergio Caravita, Gianfranco Parati, Paolo Castiglioni

**Affiliations:** 1 Istituto Auxologico Italiano, IRCCS, Department of Cardiovascular, Neural and Metabolic Sciences, S.Luca Hospital, 20149 Milan, Italy; a.faini@auxologico.it (A.F.); s.caravita@auxologico.it (S.C.); 2 Department of Management, Information and Production Engineering, University of Bergamo, 24044 Dalmine, Italy; 3 Department of Medicine and Surgery, University of Milano-Bicocca, 20126 Milan, Italy; 4 IRCCS Fondazione Don Carlo Gnocchi, 20148 Milan, Italy; pcastiglioni@dongnocchi.it

**Keywords:** Sampen, cross-entropy, autonomic nervous system, heart rate, blood pressure, hypobaric hypoxia, rehabilitation medicine

## Abstract

Stays at high altitude induce alterations in cardiovascular control and are a model of specific pathological cardiovascular derangements at sea level. However, high-altitude alterations of the complex cardiovascular dynamics remain an almost unexplored issue. Therefore, our aim is to describe the altered cardiovascular complexity at high altitude with a multiscale entropy (*MSE*) approach. We recorded the beat-by-beat series of systolic and diastolic blood pressure and heart rate in 20 participants for 15 min twice, at sea level and after arrival at 4554 m a.s.l. We estimated Sample Entropy and *MSE* at scales of up to 64 beats, deriving average *MSE* values over the scales corresponding to the high-frequency (*MSE*_HF_) and low-frequency (*MSE*_LF_) bands of heart-rate variability. We found a significant loss of complexity at heart-rate and blood-pressure scales complementary to each other, with the decrease with high altitude being concentrated at Sample Entropy and at *MSE*_HF_ for heart rate and at *MSE*_LF_ for blood pressure. These changes can be ascribed to the acutely increased chemoreflex sensitivity in hypoxia that causes sympathetic activation and hyperventilation. Considering high altitude as a model of pathological states like heart failure, our results suggest new ways for monitoring treatments and rehabilitation protocols.

## 1. Introduction

There is an increasing interest in the physiological adaptations that occur during exposures to high-altitude conditions, particularly in the alterations of autonomic cardiovascular control. This is due to the extraordinary development of mountain tourism that leads millions of people each year to stay in the high mountains for short periods of time. In addition to mountain tourism, millions of other people live permanently at high altitudes and are exposed to conditions that may cause episodes of mountain sickness [1]. The alterations to cardiovascular control caused by high altitudes are mainly due to hypobaric hypoxia, i.e., the low partial pressure of oxygen in the air, which produces an autonomic response by increasing the chemosensitivity, possibly altering the overall integrative autonomic regulation.

Interestingly, some of the cardiovascular changes that can be observed during exposure to high altitudes are similar to the autonomic alterations occurring in some diseased conditions, as in patients with heart failure, stroke or metabolic disorders [2,3]. Therefore, stays at high altitude can be viewed as an experimental model of some pathological conditions that affect autonomic cardiovascular regulation at sea level. In this regard, the study of cardiovascular control at high altitudes may help to better understand some pathophysiological mechanisms and may be beneficial for improving treatments and outcomes in rehabilitation medicine.

Most of the high-altitude studies in the literature describe the cardiovascular autonomic alterations with linear measures of heart rate variability, in relatively small groups of participants. By contrast, the literature reports very few nonlinear measures, which exclusively regard heart rate variability during sleep [4,5], when apneas induced by hypoxia may profoundly alter the heart rate dynamics. Furthermore, very few studies evaluate high-altitude alterations in beat-by-beat blood pressure variability [6,7] because of the logistical difficulties in performing such physiological recordings at high altitude. Therefore, the effect of a stay at high altitude on the complex dynamics of the cardiovascular system in the waking state remains an unexplored issue.

Our work contributes to filling this gap by assessing changes in cardiovascular complexity during a short-term stay at high altitude (4554 m a.s.l.). One of the most effective tools for extracting information on the complex dynamics of physiological systems is multiscale entropy, and our work is based on the multivariate and multiscale assessment of entropy and cross-entropy on beat-by-beat recordings of heart rate and arterial blood pressure in healthy volunteers. Our aim is to identify those aspects of the complex dynamics of the cardiovascular system that better describe the autonomic changes in response to the chemoreflex activation expected to occur in the waking state during exposure to hypobaric hypoxia. Given that high-altitude cardiovascular alterations in healthy individuals may be a model of some pathological conditions at sea level, the results of our study may indicate novel ways for monitoring the severity of a deteriorated autonomic cardiovascular control and the efficacy of treatment during rehabilitation programs.

## 2. Materials and Methods 

### 2.1. Subjects and Data Collection

This study is based on data collected previously to evaluate the effectiveness of a drug (acetazolamide) for treating mountain sickness during acute exposure to high-altitude hypoxia [8]. In the present work, we consider the 20 Caucasian volunteers of the placebo group, who completed the hemodynamic recordings at sea level and at high altitude. The placebo group was composed of 10 males and 10 females with mean (SD) age of 37 (9.5) years old and body mass index of 22.3 (2.7) kg/m^2^. They were healthy lowlanders without known cardiovascular disease or chronic cardiovascular therapy, without a history of severe mountain sickness. None of them practiced professional sports, all lived in Milan or its surroundings, and had no recent exposure to altitudes above 2000 m. They took the placebo orally in pill form twice a day.

In all participants, the cardiovascular measurements were taken twice. The first recording session was performed at baseline, in the normobaric/normoxyc conditions of our laboratory in Milan (122 m a.s.l.). The second recording session was performed in the hypobaric/hypoxic conditions of the Margherita Hut (4554 m a.s.l. on Monte Rosa in the Italian Alps). The ascent from Milan to the Margherita Hut was completed in about 28 h, by car and cable car up to 3200 m a.s.l. and then by foot, spending one night for acclimatization in the Gniffetti hut (3647 m a.s.l.). Recordings at high altitude were performed 2 days after the arrival at the Margherita Hut.

The measurements were performed in a quiet environment with ambient temperature of about 20 °C. They consisted of the simultaneous recording of one-lead electrocardiogram, ECG (PowerLab, ADInstruments, Sydney, Australia at sea level; ECG100C, MP150 Biopac Systems, Santa Barbara, CA at high altitude) and of continuous arterial blood pressure at the finger artery (Nexfin, BMEYE, Amsterdam, The Netherlands at sea level; Portapres, Finapres Medical Systems, The Netherlands at high altitude) for 15 min. Brachial arterial blood pressure waveforms were reconstructed from the measured finger blood pressure waveforms through the transfer function method [9].

Hemoglobin oxygen saturation (NPB-295, Nellcor Puritan Bennett Inc., Plaseanton, CA, USA) and the respiratory activity with a spirometry device (Vmax SensorMedics 2200, Yorba Linda, CA, USA) were also measured during the recordings. Each subject rested in a semi-recumbent position and the recordings started after an adaptation period to the new posture of at least 5 min, ensuring that the participants felt comfortable with the setup and had no apparent urges that could influence their responses. A familiarization recording session was performed several days before the first session, at sea level, which allowed the participants to become accustomed to the experimental setup (the signals acquired during the familiarization sessions are not considered in this study). At high altitude, the Lake Louise Score Questionnaires was administered to evaluate the presence (score ≥ 5) or absence of acute mountain sickness [10].

The Ethical Committee of the Istituto Auxologico Italiano (Milan, Italy) approved the study protocol (procedure number CE Auxologico: 2010_04_13_01); all the participants gave their written informed consent to the study procedures.

### 2.2. Data Preprocessing and Spectral Analysis

Recordings of finger blood pressure (BP) and of the ECG were digitized at 200 Hz and 12 bits. Each R peak of the ECG was identified by a derivative-and-threshold algorithm and a parabolic interpolation was used to refine the R wave fiducial point as suggested in [11]. The interval between consecutive R peaks, i.e., the R-R Interval (RRI), was calculated beat by beat for cardiac beats resulting from sinus node depolarization. The systolic (S) BP and diastolic (D) BP values were identified beat by beat from the finger BP signal. SBP and DBP values associated with premature beats (as identified from the ECG) or with calibrations of the device for recording finger BP were removed. The percentage of removed RRI beats was 0.5% on average, with 5% being the worst case; with respect to SBP/DBP, the percentage of removed beats increased to 2.8% on average, with 10% the worst case. The Pulse Interval (PI) was calculated beat by beat as the time interval between consecutive systolic peaks. The duration of each breathing cycle was identified on the respiratory signals as the interval between the start of consecutive inspiratory phases. The beat-by-beat series of RRI, SBP, and DBP were interpolated at 5 Hz before spectral analysis to obtain evenly sampled series and to linearly interpolate missing beats. Power spectra were calculated by the Welch periodogram with 80% overlapped Hann data windows of 240 s length and by integrating the periodogram over the very-low frequency (VLF, between 0.003 and 0.04 Hz), the low frequency (LF, between 0.04 and 0.15 Hz) and the high-frequency (HF, between 0.15 and 0.4 Hz) bands, as defined in international guidelines [11].

### 2.3. Multiscale Entropy of RRI, SBP, and DBP

We estimated the multiscale entropy (*MSE*) of RRI, SBP, and DBP with the method proposed in [12]. The method is based on the original approach to evaluate multiscale entropy as Sample Entropy, *SampEn*, of progressively coarse-grained sub-series [13] (where the coarse graining is obtained by decimation, taking one sample every *n* after a moving average of order *n*) with the same tolerance threshold at each coarse-graining order [14]. However, this includes the subsequent suggestion to replace the decimation with a delay *n* that increases the statistical consistency of the estimate [15]. Furthermore, our method substitutes the moving average with a Butterworth filter to improve the scale resolution, as shown in the case of decimation by others [16].

Briefly, given a time series of *N* samples **X** = {*x_1_,x_2_,…,x_N_*}, let’s call **Y***^n^* = {*y_1_^n^,y_2_^n^,…,y_N_^n^*} the output of the zero-phase, 6th-order low-pass Butterworth filter with cut-off frequency *f_c_* = 0.5/*n* applied to **X**. The template vectors at a given embedding dimension *m* and scale *n* are built considering a delay of *n* samples between consecutive elements:

(1) $$y_{i}^{m}(n)={\left[y_{i}^{n},y_{{}_{i+n}}^{n},\ldots ,y_{i+(m-1)n}^{n}\right]}^{T},\;1\leq I\leq N-mn$$

The infinity norm distance between any couple of template vectors is

(2) $$d_{ij}^{m}(n)={\left\|y_{i}^{m}(n)-y_{j}^{m}(n)\right\|}_{\infty },\;1\leq i,j\leq N-mn,\;j>I+n$$

and the number of “paired-vectors” *n_p_*(*m,n,r*), which are pairs of vectors with distance lower than a predefined tolerance threshold *r*, is calculated based on the infinity norm. Repeating these steps for the successive dimension *m + 1*, the sample entropy of **Y***^n^* with delay *n* and tolerance *r* is:

(3) $$SampEn\left(Y^{n},N,m,n,r\right)=-\ln\frac{n_{p}\left(m+1,n,r\right)}{n_{p}\left(m,n,r\right)}$$

This leads to the definition of the *MSE* of **X**, which is a function of the scale *n*, at a given embedding dimension *m* and tolerance *r*, as

*MSE*(*n*) = *SampEn*(**Y**^*n*^,*N*,*m*,*n*,*r*) (4)

At *n* = 1, **Y**^1^ = **X** and *MSE(1)* coincides with the *SampEn* of **X**.

For each RRI, SBP and DBP series, we calculated *MSE*(*n*) for 1 ≤ *n* ≤ 64 beats. The tolerance threshold is commonly set at *r* = 0.20 times the standard deviation of the time series in heart rate variability studies and we also adopted this choice. As to the embedding dimension, in addition to *m* = 2, traditionally used in *SampEn* analysis of heart rate variability, we also considered *m* = 1, because we previously showed that it provides *MSE(n)* profiles similar to *m* = 2 but with better statistical consistency [14]. To compare sea-level and high-altitude conditions over the same temporal scales, in seconds, we mapped the scale units from number of beats, *n*, to time *t*, in seconds, with the transformation:

*t* = *n* × <*RRI*> (5)

where *<RRI>* is the mean RRI of each series, in seconds. We interpolated and resampled *MSE* to obtain 100 estimates at scales *t* exponentially distributed over the time axis between 1 s and 48 s. This range includes the scales associated with the traditional high-frequency (HF, 2.5 ≤ *t*< 6.7 s) and low-frequency (LF, 6.7 ≤ *t* < 25 s) bands of the heart rate variability spectra. As a concise way to represent the results, the *MSE* functions were averaged over the scales included in the HF and LF bands, obtaining the *MSE_HF_* and *MSE_LF_* indices.

**Figure 1:**
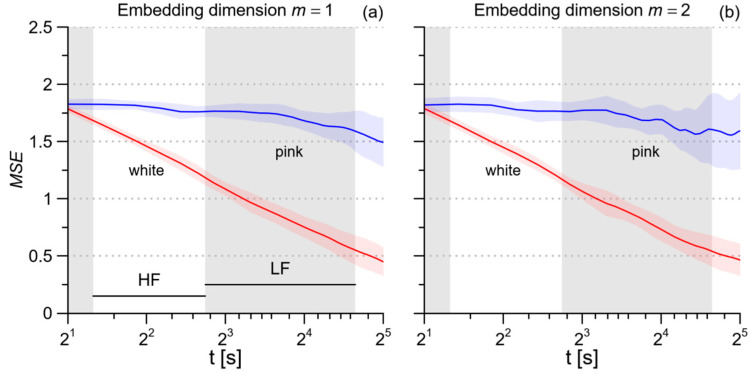
(**a**) Profiles of Multiscale Entropy MSE calculated with embedding dimension *m* = 1: mean ± SD for 10 synthesized series of white noise and 10 synthesized series of pink noise, each of 1000 samples, simulating 15’ beat-by-beat recordings with mean RRI equal to 900 ms. Gray bands show the ranges of scales corresponding to the HF and LF spectral bands. (**b**) MSE calculated with *m* = 2 for the same data of panel (**a**).

To evaluate the performance of our *MSE* estimator, we synthetized 10 series of white noise and 10 series of pink noise, each of *N* = 1000 samples. This length corresponds to 15’ recordings at the average RRI of 900 ms. Then we calculated *MSE* over the scales associated with the *HF* and *LF* bands. The estimates in Figure 1 demonstrate the capability of our MSE method to faithfully describe the entropy structures of these random processes over the scales corresponding to the HF and LF bands.

### 2.4. Multiscale Cross-Entropy between SBP and PI

To estimate the cross-entropy between blood pressure and heart rate, we used PI rather than RRI values to more easily couple blood pressure and heart rate series beat by beat (the number of valid beats of the RRI series may differ from those of SBP and PI due to the presence of calibration periods in the device measuring the finger arterial pressure). The multiscale cross-entropy, *XMSE*, between the SBP and PI series was defined extending the cross-sample entropy estimator, *XSampEn* [17], to multiple scales similarly to the way we defined *MSE* extending *SampEn* at multiple scales. However, the PI and SBP series are normalized to unit variance and zero mean before applying the Butterworth filters with cut-off frequency *f_c_* = 0.5/*n* to obtain the **P***^n^* = {*p_1_^n^,p_2_^n^ …,p_N_^n^*} and **S***^n^*={*s_1_^n^,s_2_^n^…,s_N_^n^*} output series. The template vectors for the embedding dimension *m* at scale *n* are

(6) $$\begin{array}{c} p_{i}^{m}(n)={\left[p_{i}^{n},p_{i+n}^{n},\ldots p_{{}_{i+(m-1)n}}^{n}\right]}^{T}\\ s_{i}^{m}(n)={\left[s_{i}^{n},s_{i+n}^{n},\ldots s_{{}_{i+(m-1)n}}^{n}\right]}^{T} \end{array},\;1\leq I\leq N-mn$$

Based on the distances between couples of vectors

(7) $$d_{ij}^{m}(n)={\left\|p_{i}^{m}(n)-s_{j}^{m}(n)\right\|}_{\infty },\;1\leq ij\leq N-mn$$

the number of paired vectors with distance lower than a threshold *r*, *n_px_*(*m,n,r*), is calculated. Repeating these steps for *m* + *1*, the cross-*SampEn* between **P***^n^* and **S***^n^* with delay *n* is:

(8) $$XSampEn(P^{n},S^{n},N,m,n,r)=-\ln\frac{n_{px}\left(m+1,n,r\right)}{n_{px}\left(m,n,r\right)}$$

and the multiscale cross-entropy between SBP and PI is estimated as:

*XMSE*(*n*) = *XSampEn*(**P**^*n*^,**S**^*n*^,*N*,*m*,*n*,*r*) (9)

*XMSE* between SBP and PI was assessed at the embedding dimensions *m* = 1 and *m* = 2, with *r* = 0.20. Clearly, at *n* = 1 *XMSE* coincides with the cross-SampEn. The scales were mapped from beats, *n*, into times, τ, according to Equation (5), then interpolated and resampled in a similar fashion between 1 and 48 s. Finally, the *XMSE* functions were averaged over the scales included in the HF and LF bands, obtaining the *XMSE_HF_* and *XMSE_LF_* indices.

### 2.5. Statistical Analysis

All indices were compared between sea level and high altitude with paired t-tests whenever their distribution passed the Shapiro-Wilks Gaussianity test at p = 0.05, possibly after a Box-Cox transformation [18]. Otherwise, they were compared by the non-parametric Wilcoxon signed-rank test. As regards spectral analysis, powers were log-transformed and we considered only participants with an average breathing period shorter than 7 s because the HF band correctly reflects respiratory-driven modulations only in this case; for this reason, we discarded 5 participants from the statistical tests on spectral powers.

Furthermore, we calculated the Wilcoxon signed-rank test statistics of multiscale entropy at each scale separately to easily identify the scales better reflecting the alterations induced by high altitude in the *MSE*(*t*) and *XMSE*(*t*) profiles. With respect to the derived entropy indices (namely *SampEn*, *MSE_HF,_* and *MSE_LF_* of RRI, SBP, and DBP; *XSampEn*, *XMSE_HF_*, and *XMSE_LF_* between SBP and PI) we also calculated the 95% confidence intervals of the mean for the difference between high-altitude and sea-level conditions. The confidence intervals were obtained with the nonparametric bootstrap method by randomly sampling with replacement the original 20 measures 1000 times; this bootstrapping allows high-quality estimates of the confidence intervals to be obtained with a distribution-free approach that avoids making any assumption on the nature of the distributions.

The threshold for statistical significance was set at 5%. with a two-sided alternative hypothesis. All the statistical tests were performed with “R: A Language and Environment for Statistical Computing” software package (R Core Team, R Foundation for Statistical Computing, Vienna, Austria, 2018).

## 3. Results

The high-altitude conditions were characterized by faster and deeper breathing, by lower hemoglobin oxygen saturation, and by higher blood pressure and heart rate levels (Table 1). The spectral powers of RRI changed at high altitude, with decreased VLF, LF and HF powers and increased LF/HF powers ratio. By contrast, the high altitude did not change the LF and HF powers of SBP and DBP and only marginally decreased their VLF power (Table 1). Half of the participants (N = 10, 6 males) presented acute mountain sickness.

### 3.1. Multiscale Entropy

**Figure 2:**
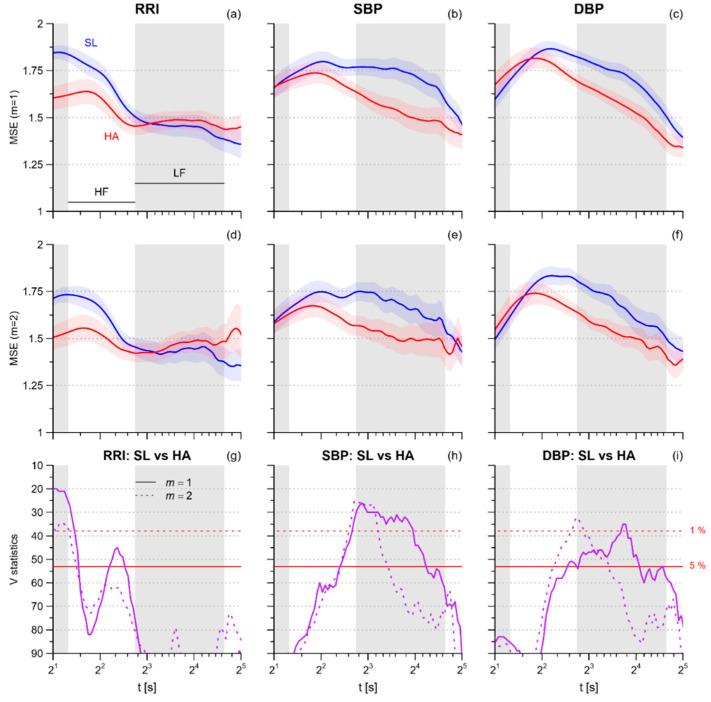
(**a**) Profiles of Multiscale Entropy MSE at sea level (SL, blue lines) and high altitude (HA, red lines) for RRI calculated with embedding dimension *m* = 1: mean ± sem on 20 participants (gray bands show the ranges of scales corresponding to the HF and LF spectral bands); (**b**) MSE calculated as in (**a**) for SBP; (**c**) MSE calculated as in (**a**) for DBP; (**d**) MSE for RRI calculated as in (**a**) but with *m* = 2; (**e**) MSE calculated as in (**d**) for SBP; (**f**) MSE calculated as in (**d**) for DBP; (**g**) Wilcoxon signed-rank statistics *V* for the comparison between SL and HA for MSE of RRI; the red horizontal lines are the 5% (continuous) or 1% (dashed) percentiles of the distribution for the null hypothesis: when *V* is above these thresholds the hypothesis of similar entropies can be rejected at the corresponding significance level; (**h**) *V* statistics for the comparison between SL and HA for SBP MSE; (**i**) *V* statistics for the comparison between SL and HA for DBP MSE.

Figure 2 compares the profiles of multiscale entropy. At sea level, the *MSE(t)* profile of RRI decreases with *t* from the maximum at 2 s, reaching a plateau at scales greater than 7 s. The high-altitude condition reduces *MSE* at the shorter end only, and thus the *MSE(t)* profile is flatter at high altitude. By contrast, SBP and DBP have higher *MSE* values at scales within the HF band and this pattern is more pronounced at high altitude due to the substantial reduction of *MSE* at scales within the LF band; in particular, at scales greater than 10 s, the entropy reduction is more significant for *m* = 1.

*SampEn*, which corresponds to *MSE* at the scale of 1 beat, decreases at high altitude for RRI only (Table 2), without changes for SBP or DBP. The decrease is more significant for *m* = 2.

Similarly, we found a significant reduction of *MSE_HF_* for RRI and not for SBP or DBP; by contrast, *MSE_LF_* decreased at high altitude for SBP and DBP but not for RRI, in these cases with greater statistical significance for *m* = 1 (Table 3).

**Figure 3:**
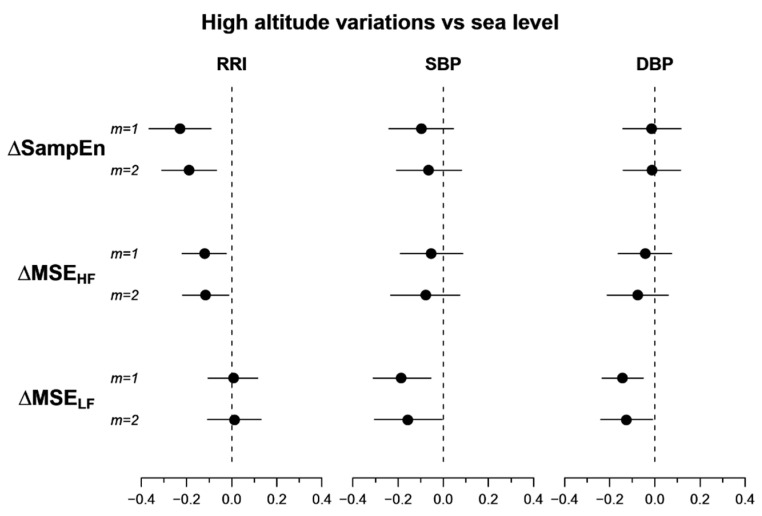
95% confidence intervals of the difference between high-altitude and sea-level conditions of entropy indices. *From top to bottom*: Sample Entropy (*SampEn*), multiscale entropy over the HF (*MSE_HF_*) and over the LF (*MSE_LF_*) bands; *m* is the embedding dimension.

The 95% confidence intervals of the differences between high altitude and sea level (Figure 3) confirm that exposure to high altitude reduces *SampEn* and *MSE_HF_* of RRI and reduces *MSE_LF_* of SBP and DBP. The greater statistical power of the bootstrap method in Figure 3 compared to the paired *t*-test in Table 2 is reflected in the 95% confidence intervals of the *SampEn* variations which do not cross the zero line also for *m* = *1*.

### 3.2. Multiscale Cross-Entropy

**Figure 4:**
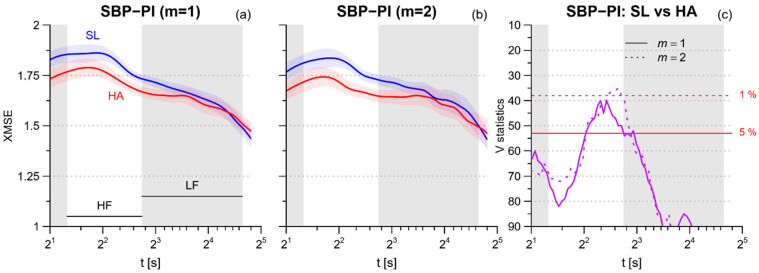
(**a**) Profiles of multiscale cross-entropy XMSE between SBP and PI at sea level (SL, blue lines) and high altitude (HA, red lines): mean ± sem for embedding dimension *m* = 1; (**b**) XMSE from the same data of panel (**a**) calculated for embedding dimension *m* = 2; (**c**) Wilcoxon signed-rank statistics *V* for the comparison between SL and HA; the red horizontal lines are the 5% (continuous) or 1% (dashed) percentiles of the distribution for the null hypothesis: when *V* is above these thresholds, the hypothesis of similar entropies can be rejected at the corresponding significance level. Gray bands show the ranges of scales corresponding to the HF and LF spectral bands.

The scale-by-scale profiles of *XMSE* between SBP and PI (Figure 4) show the highest values at scales within the HF band. The high altitude decreases cross-entropy at the shorter scales, the reduction being significant at 3–4 s.

**Figure 5:**
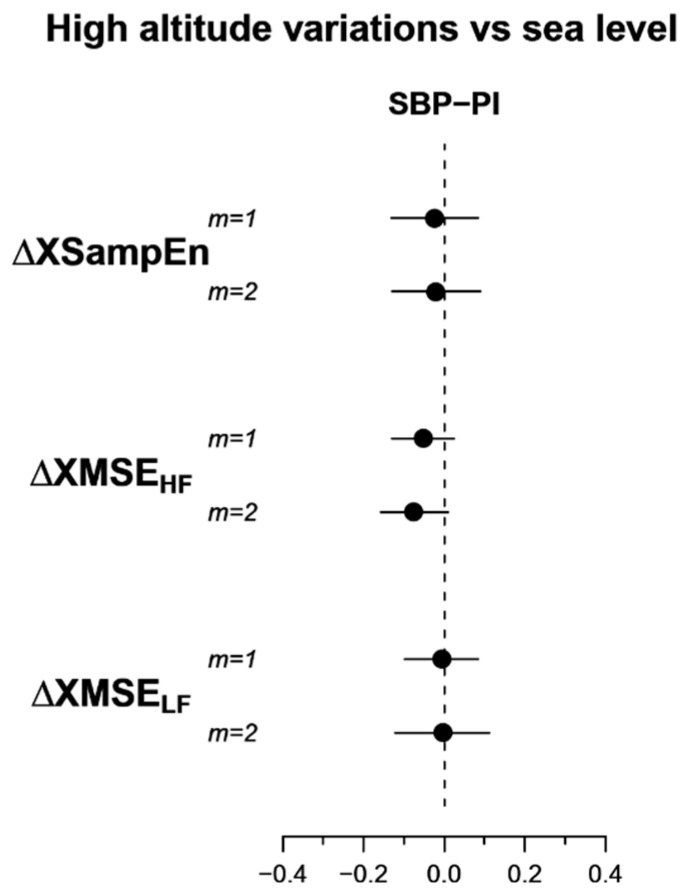
95% confidence intervals of the difference between high-altitude and sea-level conditions of cross-entropy indices. *From top to bottom*: Cross sample entropy (*XSampEn*), cross multiscale entropy over the HF (*XMSE_HF_*) and the LF (*XMSE_LF_*) bands; *m* is the embedding dimension.

However, the average decrease of SBP-PI cross-entropy in the HF band, *XMSE_HF_,* does not reach the statistical significance; furthermore, *XSampen* and *XMSE_LF_* appear substantially similar in the two conditions (Table 4 and Figure 5).

## 4. Discussion

We described the effects of a short stay at high altitude on the complex dynamics of the cardiovascular system. Novelties of our study are that for the first time, the effects of high altitude are described with a multiscale entropy approach, and that the cardiovascular dynamics is evaluated considering not only the heart rate variability, as is usually done in studies on the autonomic cardiovascular control at high altitude, but also the beat-by-beat variability of arterial blood pressure. It should be noted that traditional spectral analysis and complexity analysis (when assessed as multiscale entropy) investigate very different aspects of the time series dynamics, with one being related to the amplitude of the fluctuations, and the other to their unpredictability/irregularity. The entropy approach (as originally proposed by Costa et al. [13,19] and which we follow even if using a different, statistically more consistent, estimator for short series) assumes that a proper decomposition of the entropic measure of irregularity at different temporal scales reveals the capability of dynamical systems to adapt to external perturbations and to environmental changes. Following this proposal, multiscale entropy has been assessed in several studies aimed at quantifying the loss of complexity in diseased states, as a way to assess the reduced adaptive capacity of the individual. Similarly, we applied multiscale entropy to quantify the degraded adaptive capacities of the cardiovascular system exposed to high altitude conditions. Interestingly, our results represent an example of the different information provided by spectral analysis and by entropy-based complexity analysis.

### 4.1. Cardiorespiratoy Variables and Spectral Powers at High Altitude

Our participants showed the marked decrease of hemoglobin oxygen saturation expected at such a high altitude and the hyperventilation triggered by the chemoreflex control of breathing in response to hypoxia [20]. Acute hypoxia also stimulates peripheral chemoreceptors, producing a sympathetic activation that increases blood pressure [21,22], explaining the increased blood pressure levels we observed at high altitude. The sympathetic activation could also explain the higher heart rate and LF/HF spectral measures, index of cardiac sympatho/vagal balance; furthermore, it could have induced a general decrease of the vagal modulations of heart rate, as quantified by the reduced HF and LF powers. These spectral changes are in line with those repeatedly reported on the acute autonomic effects after an ascent at high altitude [23,24,25,26] or at a simulated altitude of 3600 m asl in a hypobaric/hypoxic chamber [27,28]. By contrast, an increased LF power without HF power changes [5] was reported at 3600 m asl; however, differently from our study and from [23], recordings were performed during nighttime sleep when periodic breathing and apneas are likely to occur, making the HF power an unreliable index of the vagal respiratory modulations of the heart rate. 

### 4.2. Heart Rate Multiscale Entropy

The multiscale entropy of heart rate is a measure of the complexity of the cardiovascular system [13]. The shortest possible scale at which the multiscale entropy can be calculated is the single beat, and in this case, *MSE* coincides with SampEn. Maneuvers eliciting the sympatho/vagal balance, like posture changes or pharmacological blockade [29,30], decrease the heart-rate SampEn, and thus the significant reduction of RRI SampEn at high altitude (Figure 3) could be a consequence of the sympathetic activation induced by the acute exposition to high-altitude hypoxia. Coherently with our result, a decrease of the heart-rate SampEn was observed during simulated high altitude [27], as well as in a real high-altitude environment [5,26]. The opposite trend was reported in participants suddenly exposed to hypoxia simulating the altitude of 8230 m asl in a hypobaric chamber [31], a result that could reflect an abrupt activation of a defensive autonomic response caused by the sudden exposure to such an extreme condition.

However, the novel results of our study are the *MSE* alterations at scales greater than 1 beat. We found a significant decrease in *MSE* over the scales of the HF band that does not extend to larger scales (Figure 2 and Figure 3, Table 2). This suggests a loss of cardiovascular complexity that mainly affects the faster components, probably associated with ventilation, while the cardiac complexity at longer scales is preserved. For some aspects, like the sympathetic and ventilatory responses to hypercapnia [32], the high-altitude condition may represent a model of heart failure and it is worth noting that heart failure patients, compared to healthy subjects, have lower *MSE* values over a broad range of scales that includes the HF band [33].

### 4.3. Blood Pressure Multiscale Entropy

Another novel finding regards the alterations in the *MSE* of blood pressure. While exposure to high altitude reduces the *MSE* of RRI in the HF band and at shorter scales, it reduces the *MSE_LF_* of SBP and DBP without affecting their SampEn or *MSE_HF_*. The lack of alterations in the faster components of blood pressure complexity could be related to the non-autonomic nature of the blood pressure dynamics at scales faster than the LF band. For instance, HF modulations of blood pressure are mainly due to the direct action of the respiratory mechanics, and not to autonomic modulations mediated by chemo- or baro-reflexes, as for RRI. Interestingly, an autonomic influence on blood pressure is expected over a range of larger scales that includes the LF band. At these scales, the sympathetic outflow that reaches the individual vascular districts modulates the arteriolar resistances in order to regulate the local supplies of blood. It could be possible that the high-altitude hypoxia induced overall sympathetic vasoconstrictions to make more oxygen available to the brain and the heart, and that the vasoconstriction substantially decreased the amplitude of local vasomodulations. Therefore, the observed loss of blood pressure complexity in the LF band might reflect an altered sympathetic control of local vascular districts.

### 4.4. Blood Pressure-Heart Rate Multiscale Cross-Entropy

We estimated cross-entropy with the *XMSE* estimator to evaluate the degree of asynchronicity between blood pressure and heart rate. We used PI values rather than RRI values to more easily couple the blood pressure and heart rate beat by beat. *XMSE* is based on the conditional probability that SBP-PI pairs of segments that are similar when observed over *m* beats remain similar when the segments are increased by one beat (the higher the probability, the lower the cross-entropy). *XMSE* can therefore provide a more general assessment of the synchronization between time series than other analysis tools, like the squared coherency spectrum, which may reflect the linear components only of the coupling between time series. 

Even if *MSE* decreased substantially for RRI at the faster scales and for SBP at the lower scales, the *XMSE* between SBP and PI showed only a marginal decrease at scales around 5 s with a non-significant reduction of *XMSE_HF_*. This would indicate that the level of synchronization between the two series is preserved, if not even slightly increased in the HF band. A possible explanation for this trend is related to the mechanism that couples SBP with PI in the HF band, i.e., respiration. Each inspiratory phase increases the filling of the left ventricle and thus the stroke volume of the following beat, which in turn increases SBP. The increase in SBP is sensed by the baroreceptors and triggers a vagal baroreflex response that lengthens the following cardiac interval. In this way, a respiratory oscillation is mechanically generated in SBP and coupled to a baroreflex-mediated oscillation in PI, with the same frequency. The minute ventilation increased dramatically at high altitude (Table 1), and thus it may be responsible for SBP and PI coupled oscillations with larger amplitude, and thus for the increased SBP-PI synchronization in the HF band.

### 4.5. Limitations and Conclusion

Cardiorespiratory control adapts differently in males and females to short-term stays at high altitude [2,3], and although a recent study did not report an interaction with sex in the effect of high altitude on spectral indices of heart rate variability [34], it cannot be excluded that the alterations of multiscale entropy we described are gender dependent. Our participants were matched by sex (10 males and 10 females), so our results are not biased by the gender composition. However, a larger population is needed to stratify our results by sex.

Another factor possibly influencing the cardiac autonomic adaptation to high altitude is the presence of acute mountain sickness [23]. Our group was composed of 10 participants with acute mountain sickness (age 37.9 ± 8.9 years old) and 10 without acute mountain sickness (age 35.6 ± 9.6 years old), and the results should reflect those of a general population ascending to similar high altitudes [35]. However, a larger group of participants is required to evaluate the possible influence of acute mountain sickness on cardiovascular complexity.

Genetic factors [36,37] and acclimatization [38] may play a role in the capability of the autonomic cardiovascular control to adapt to high-altitude environments. Since all our participants were lowlanders belonging to the same Caucasian ethnicity, and with no recent exposure to high altitude, our study was not designed to investigate the possible influence of genetic factors or acclimatization. Larger groups are needed to evaluate whether similar alterations in multiscale entropy may also characterize different ethnicities or highlander populations. Cardiorespiratory diseases may produce alterations in cardiovascular control even during short-term exposure to moderate altitudes [39]. Since we included only healthy volunteers without known cardiovascular diseases or chronic therapies, future studies are needed to evaluate whether the alterations we observed may be exacerbated by diseased conditions.

We had to use different measuring devices at sea level and at high altitude due to organizational reasons, and in theory, this might have influenced our results. However, since sampling rates, digital resolution, and analog preprocessing filters were the same, we can exclude differences in the quality of the ECG recordings. As to the finger blood pressure, the measuring device at high altitude (Portapres, Finapres Medical Systems, The Netherlands), although specifically designed for portability, is based on the same physical principles and technologies of the laboratory device used at sea level (Nexfin, BMEYE, Amsterdam, The Netherlands). Therefore, we can exclude differences due to the quality of the measuring devices also for the BP measures.

In conclusion, we assessed the alterations induced by a short stay at high altitude in the complexity of the cardiovascular system with a multiscale entropy approach. The alterations indicate a loss of complexity at specific ranges of scales that differ between heart rate and blood pressure and are complementary to each other, being the complexity loss concentrated at the shorter scales for heart rate and at the longer scales for blood pressure. The changes can be ascribed to the increased chemoreflex sensitivity in hypoxia that causes sympathetic activation and hyperventilation. These results may contribute to understanding the physiological adaptations to high altitude; furthermore, considering high-altitude conditions as a model of pathological states like heart failure, they may also help to better understand the loss of cardiovascular complexity in patients, possibly suggesting effective ways to improve treatments or to monitor rehabilitation protocols.

## Figures and Tables

**Table 1:** General cardiorespiratory characteristics and spectral indices: mean (SD) at sea level and high altitude.

	Sea Level	High Altitude	*p* Value
Respiration			
*breathing rate* (bpm)	12.1 (4.4)	16.9 (6.3) **	<0.001
*minute ventilation* (L/min)	6.7 (1.4)	11.0 (2.7) **	<0.001
*oxygen saturation* (%)	97.6 (1.1)	77.4 (6.7) **	<0.001
RRI			
*mean* (ms)	956.4 (120.4)	770.2 (95.2) **	<0.001
*Total power* (ms^2^)	5490 (5309)	2114 (1631) **	<0.001
*VLF power* (ms^2^)	2146 (2265)	929 (736) **	0.001
*LF power* (ms^2^)	1711 (1546)	576 (453) **	<0.001
*HF power* (ms^2^)	1233 (1270)	403 (516) **	<0.001
*LF/HF powers ratio*	2.11 (1.68)	3.11 (3.03) *	0.05
SBP			
*mean* (mmHg)	109.5 (13.7)	120.1 (10.3) **	0.002
*Total power* (mmHg^2^)	39.82 (22.60)	26.12 (16.57)	0.10
*VLF power* (mmHg^2^)	24.06 (16.96)	12.16 (8.46)	0.06
*LF power* (mmHg^2^)	11.01 (6.10)	8.82 (4.78)	0.40
*HF power* (mmHg^2^)	2.36 (1.17)	3.28 (4.45)	0.40
DBP			
*mean* (mmHg)	74.8 (9.4)	80.7 (9.9) **	0.001
*Total power* (mmHg^2^)	18.60 (13.17)	13.77 (13.77)	0.10
*VLF power* (mmHg^2^)	10.52 (9.28)	6.21 (6.95) *	0.040
*LF power* (mmHg^2^)	6.23 (4.02)	5.65 (4.68)	0.30
*HF power* (mmHg^2^)	0.84 (0.56)	0.89 (1.23)	0.50

* and ** indicate differences at 5% and 1% significance; 5 of the 20 participants are discarded from the statistics on spectral powers because their average breathing rate felt below the HF band.

**Table 2:** *SampEn*: mean (SD) over the group at sea level and high altitude.

	Sea Level	High Altitude	*p* Value
RRI			
*m* = 1	1.57 (0.18)	1.34 (0.37)	0.06
*m* = 2	1.39 (0.23)	1.21 (0.35) **	0.007
SBP			
*m* = 1	1.35 (0.28)	1.25 (0.25)	0.2
*m* = 2	1.22 (0.28)	1.15 (0.25)	0.4
DBP			
*m* = 1	1.28 (0.25)	1.26 (0.25)	0.8
*m* = 2	1.19 (0.25)	1.17 (0.24)	0.9

*m* = embedding dimension; ** indicates differences at 1% significance.

**Table 3:** Multiscale entropy over the HF and LF band: mean (SD) at sea level and at high altitude.

		*MSE_HF_*			*MSE_LF_*	
	Sea Level	High Altitude	*p* Value	Sea Level	High Altitude	*p* Value
RRI						
*m* = 1	1.69 (0.20)	1.57 (0.25) *	0.031	1.46 (0.24)	1.47 (0.21)	0.9
*m* = 2	1.62 (0.22)	1.50 (0.26) *	0.043	1.45 (0.28)	1.46 (0.24)	0.8
SBP						
*m* = 1	1.76 (0.25)	1.71 (0.22)	0.08	1.72 (0.26)	1.53 (0.25) *	0.014
*m* = 2	1.71 (0.26)	1.64 (0.25)	0.09	1.67 (0.31)	1.51 (0.28)	0.06
DBP						
*m* = 1	1.82 (0.21)	1.78 (0.21)	0.5	1.70 (0.20)	1.56 (0.17) **	0.009
*m* = 2	1.78 (0.23)	1.71 (0.23)	0.3	1.65 (0.26)	1.52 (0.19)	0.06

*m* = embedding dimension; * and ** indicate differences at 5% and 1% significance.

**Table 4:** SBP-PI cross-entropy indices: mean (SD) at sea level and at high altitude.

	Sea Level	High Altitude	*p* Value
*XSampEn*			
*m* = 1	1.57 (0.19)	1.54 (0.26)	0.7
*m* = 2	1.50 (0.23)	1.47 (0.25)	0.7
*XMSE_HF_*			
*m* = 1	1.82 (0.16)	1.77 (0.16)	0.2
*m* = 2	1.79 (0.16)	1.72 (0.19)	0.10
*XMSE_LF_*			
*m* = 1	1.63 (0.16)	1.63 (0.15)	>0.9
*m* = 2	1.63 (0.19)	1.62 (0.19)	>0.9

*m* = embedding dimension.
